# Correlates of Trabecular and Cortical Volumetric Bone Mineral Density of the Radius and Tibia in Older Men: The Osteoporotic Fractures in Men Study

**DOI:** 10.1002/jbmr.6

**Published:** 2009-12-21

**Authors:** Kamil E Barbour, Joseph M Zmuda, Elsa S Strotmeyer, Mara J Horwitz, Robert Boudreau, Rhobert W Evans, Kristine E Ensrud, Moira A Petit, Christopher L Gordon, Jane A Cauley

**Affiliations:** 1 Department of Epidemiology, Graduate School of Public Health, University of Pittsburgh Pittsburgh, PA, USA; 2 Division of Endocrinology and Metabolism, University of Pittsburgh School of Medicine Pittsburgh, PA, USA; 3 Department of Rheumatology, Minneapolis VA Medical Center Minneapolis, MN, USA; 4 School of Kinesiology, University of Minnesota Minneapolis, MN, USA; 5 Department of Radiology, McMaster University Hamilton, Ontario, Canada

**Keywords:** osteoporosis, vBMD, pQCT, radius, tibia

## Abstract

Quantitative computed tomography (QCT) can estimate volumetric bone mineral density (vBMD) and distinguish trabecular from cortical bone. Few comprehensive studies have examined correlates of vBMD in older men. This study evaluated the impact of demographic, anthropometric, lifestyle, and medical factors on vBMD in 1172 men aged 69 to 97 years and enrolled in the Osteoporotic Fractures in Men Study (MrOS). Peripheral quantitative computed tomography (pQCT) was used to measure vBMD of the radius and tibia. The multivariable linear regression models explained up to 10% of the variance in trabecular vBMD and up to 9% of the variance in cortical vBMD. Age was not correlated with radial trabecular vBMD. Correlates associated with both cortical and trabecular vBMD were age (−), caffeine intake (−), total calcium intake (+), nontrauma fracture (−), and hypertension (+). Higher body weight was related to greater trabecular vBMD and lower cortical vBMD. Height (−), education (+), diabetes with thiazolidinedione (TZD) use (+), rheumatoid arthritis (+), using arms to stand from a chair (−), and antiandrogen use (−) were associated only with trabecular vBMD. Factors associated only with cortical vBMD included clinic site (−), androgen use (+), grip strength (+), past smoker (−), and time to complete five chair stands (−). Certain correlates of trabecular and cortical vBMD differed among older men. An ascertainment of potential risk factors associated with trabecular and cortical vBMD may lead to better understanding and preventive efforts for osteoporosis in men. © 2010 American Society for Bone and Mineral Research.

## Introduction

There is growing recognition that osteoporosis and fractures in older men are significant public health problems that contribute to disability and premature death.(1) Osteoporosis is defined as a systemic bone disease characterized by low bone mass and mircoarchitectural deterioration of bone tissue, with a subsequent increase in bone fragility and susceptibility to fracture.(2) Low bone mineral density (BMD) is an important risk factor for fractures in men. Men over the age of 50 have a 13% estimated risk of developing an osteoporotic fracture.(3)

A major limitation of dual-energy X-ray absorptiometry (DXA) is its inability to distinguish cortical and trabecular bone. Bones tend to differ in their composition of trabecular and cortical bone. For example, the spine is primarily trabecular bone.(4) Therefore, identifying correlates of trabecular bone can lead to a better understanding of what factors might be associated with spine fracture. Also, DXA provides a measurement in only two dimensions and thus reports an areal BMD (aBMD). Bones of larger width and length tend to have greater depth (eg, in men compared with women), and since bone depth is not accounted for in DXA scanning, reliance on aBMD inherently underestimates bone density.(5) Volumetric BMD (vBMD) has been shown to be a stronger predictor of vertebral fractures than aBMD in some studies.(6,7) Quantitative computed tomography (QCT) assesses vBMD and distinguishes cortical from trabecular bone.(8) The correlates of DXA-measured BMD have been well established in men.(1,9–13) However, few studies have examined the correlates of vBMD, especially in older men.(8,11) The aim of this study was to identify correlates of trabecular and cortical vBMD using peripheral QCT (pQCT) and examine whether they differ.

## Materials and Methods

### Study population

From March 2000 through April 2002, 5995 men aged 65 years or older were recruited using population-based listings from six US clinical sites to participate in the Osteoporotic Fractures in Men Study (MrOS).(14,15) pQCT measurements were obtained at the Minneapolis, MN, and Monongahela Valley, PA, study sites during the second clinic visit between March 21, 2005, and April 11, 2006, for 1172 individuals (46.1% from MN and 53.9% from PA) aged 69 to 97 years. The institutional review board at each center approved the study protocol, and written informed consent was obtained from all the participants.

### pQCT measurements

pQCT examinations were performed on the Sratec XCT scanner series (2000 or 3000, Stratec, Inc., Pforzheim, Germany). Trabecular vBMD of the radius and tibia was measured by obtaining an ultradistal slice at 4% of the length of the ulna proximal to the radial endplate and at 4% of the tibia length proximal to the tibial endplate, respectively. Cortical vBMD of the radius and tibia was measured by obtaining a distal slice at 33% of the length of the ulna proximal to the radial endplate and at 33% of the tibia length proximal to the tibial endplate, respectively. A scout view (an anatomic reference line for the relative location of subsequent scans) was obtained prior to the pQCT scan at the tibia and radius. Precise assessment of bone mineral properties by pQCT was ensured by minimizing participant movement. A quality assurance (QA) phantom scan was used to monitor the stability of the pQCT scanner. A cross-calibration check also was performed between the centers. The European Forearm Phantom was scanned three times at each site at 200, 100, and 50 mg/mL, respectively. Voxel size was 0.5 mm, and the scan speed was 25 mm/s. To determine the trabecular vBMD(mg/cm^3^), all radius and tibia scans were analyzed using identical parameters for contour findings and separation of trabecular and cortical bone (contour mode 2, *T* = 169 mg/cm^3^; peel mode 1, area = 45%). All proximal radius and tibia shaft scans were analyzed using identical parameters for contour finding and separation of total and cortical bone (contour mode 2, *T* = 169 mg/cm^3^; cortmode 1, *T* = 710 mg/cm^3^) to determine the vBMD of the cortical-rich bone compartment (mg/cm^3^). Coefficients of variation (CVs) were determined for pQCT scans by replicating measurements on 15 subjects (CV ≤ 2.1%).(8)

### Other measurements

Self-administered and interviewer-administered questionnaires were used by trained clinical staff to obtain demographic, medical, and lifestyle information from the participants. Information on anthropometric measures, neuromuscular function, and medication use also was obtained at the clinic.(14) Information on covariates was obtained from visit 2, with the exception of race/ethnicity, education, alcohol intake, calcium and vitamin D intake, history of a nontrauma fracture after age 50, self-report of diabetes and fasting glucose level, whether a subject ever had a gastrectomy, and testosterone injection use, all of which was abstracted at baseline.

Race/ethnicity was based on self-declaration and included the following categories: white, African American, Hispanic, Asian, and other. The participants were divided into two categories of education for analysis: greater than a high school education and high school education or lower.

A calibrated balance-beam scale was used to measure weight in kilograms. Participants had to wear indoor clothing and remove their shoes when their weight was being measured. Height was measured in meters using a Harpenden Stadiometer (Dyfed, UK).

Gait speed (m/s) was determined as the time to complete a 6-m walk. Grip strength (kg) was measured twice using a handheld Jamar dynamometer (Sammons Preston Rolyan, Bolingbrook, IL, USA) and taking the average in both the right and left arms.(16) Time to complete five chair stands (seconds) and ability to stand from a chair without using the arms (yes/no) also were measured.

Lifestyle factors included self-report of smoking (current, past, and never), alcohol intake (drinks/week), caffeine intake (mg/day), and time spent walking (hours/day). Overall physical activity also was measured by computing the physical activity summary scale for the elderly (PASE).(17) The modified Block Food Frequency Questionnaire was used to obtain dietary information related to calcium and vitamin D intake over the past year.(18)

Subjects were asked to report if they were ever told by a doctor or health care provider that they had certain medical conditions, including hypertension, stroke, myocardial infarction (MI), chronic obstructive pulmonary disease (COPD), cancer, osteoporosis, osteoarthritis (OA), hyperthyroidism, hypothyroidism, Parkinson's disease, and kidney stones. Men were classified as having diabetes if they had glucose levels ≥ 126 mg/dL (after a minimum of an 8-hour fast) at baseline, made a self-report of diabetes at baseline or visit 2, or were taking insulin or hypoglycemic medications at baseline or visit 2. Men with diabetes were further categorized as having a history of thiazolidinedione (TZD) versus non-TZD use. We are unable to distinguish type 1 and 2 diabetes. However, based on the older age of the participants and the shorter longevity associated with type 1 diabetes for this birth cohort, it is likely that nearly all have type 2 diabetes.

Participants were asked to bring all current (use within last 30 days) prescription and nonprescription medications with them to clinic. Interviewers completed a medication history for each participant, including name of medication and frequency of use. All prescription medications recorded by the clinics were stored in an electronic medications inventory database (San Francisco Coordinating Center, San Francisco, CA, USA). Each medication was matched to its ingredient(s) based on the Iowa Drug Information Service (IDIS) Drug Vocabulary (College of Pharmacy, University of Iowa, Iowa City, IA, USA).(19) Androgen use was defined as testosterone or dehydroepiandrosterone (DHEA) use. Antiandrogen use included use of flutamide or bicalutamide.

### Statistical analysis

All statistical analyses were performed using the Statistical Analysis System (SAS, Version 9.1, SAS Institute, Cary, NC, USA). Analysis of variance (ANOVA) was used to compare the unadjusted skeletal site-specific vBMD across age groups with a test of trend and a Bonferroni adjustment for pairwise comparisons. Linear regression analyses for age- and age- and weight-adjusted models were used to examine the association of each correlate with cortical and trabecular vBMD at the radius and tibia. The associations were expressed as a 1 unit increase for categorical variables and 1 standard deviation (SD) increase for continuous variables. The formula used to calculate the percent difference in vBMD per unit change of predictor variable was (β coefficient × unit/mean vBMD) × 100. The corresponding confidence intervals were calculated as [(β coefficient ± 1.96 × standard error) × (unit)/(mean vBMD)] × 100. Variables with a *p* value of less than .10 from the age- and weight-adjusted models were entered into the multivariable models. We excluded men from the multivariable analysis who reported to be taking osteoporosis medication (*n* = 42) or self-reported osteoporosis (*n* = 41) because these men, on average, had markedly lower vBMD values compared with nonusers. For the multivariable models, we used the backward elimination procedure at each skeletal site with age forced in the models. A stepwise selection procedure produced similar results. Multicollinearity was assessed using the variance inflation factor (VIF). An additional analysis examined whether correlates of vBMD differed after excluding nonwhite men in the multivariable analysis. To further understand whether the association between diabetes and vBMD is independent or modified by hypoglycemic medication use, we performed secondary multivariable analyses with and without, taking into account diabetic medications. For the diabetic medications parameter, men were categorized as nonusers of hypoglycemic medication, users of insulin or hypoglycemic agents without a history of TZD use, and use of any TZD either with or without additional hypoglycemic medications.

## Results

The study population consisted of whites (97.9%), African Americans (1.4%), Asians (0.2%), Hispanics (0.3%), and others (0.2%). The average age of the men was 77.2 ± 5.1 years, and 61.9% had higher than a high school education. The mean and SD values for trabecular and cortical vBMD were 197 ± 46 and 1159 ± 34 mg/cm^3^, respectively, for the radius and were 231 ± 41 and 1136 ± 35 mg/cm^3^, respectively, for the tibia. Tables 1 and 2 show the age- and age- and weight-adjusted models. These results were used to build the multivariable models.

**Table 1:** Percent Difference in Trabecular and Cortical vBMD of the Radius per Unit Change in Potential Correlates for Age- and Age- and Weight-Adjusted Models

			Percent difference in vBMD per unit change (95% CI)			
			
Variable	Mean ± SD or prevalence	Unit^a^	Age-adjusted trabecular vBMD	Age- and weight-adjusted trabecular vBMD	Age-adjusted cortical vBMD	Age- and weight-adjusted cortical vBMD
*Demographics*						
Age (years)	77.2 ± 5.1	5.1	−0.8 (−2.1, 0.6)^b^	−0.3 (−1.8, 1.1)	−0.5 (−0.7, −0.3)*	−0.6 (−0.5, −0.1)*
Race						
White		97.9%	—	—	—	—
African-American		1.4%	−8.3 (−20.3, 3.7)	−7.8 (−19.8, 4.1)	−0.9 (−2.4, 0.5)	−1.0 (−2.5, 0.4)
Asian		0.2%	−19.2 (−51.7, 13.3)	−16.7 (−49.3, 15.9)	−0.9 (−3.1, 5.0)	0.4 (−3.7, 4.4)
Hispanic		0.3%	−11.6 (−34.7, 11.4)	−12.2 (−35.2, 10.8)	−0.7 (−3.6, 2.1)	−0.6 (−3.5, 2.2)
Other		0.2%	18.1 (−14.5, 50.6)	18.0 (−14.5, 50.5)	3.9 (−1.2, 7.9)**	3.9 (−0.1, 7.9)**
High school education		61.9%	−0.3 (−3.1, 2.5)	−0.3 (−3.1, 2.6)	0.5 (0.1, 0.8)*	0.5 (0.1, 0.8)*
*Site*						
Monongahela Valley, PA		53.9%	−0.3 (−3.1, 2.4)	0.3 (−3.0, 2.5)	−0.3 (−0.1, 0.6)	−0.3 (−0.1. 0.6)
*Anthropometry*						
Weight (kg)	83.9 ± 13.5	13.5	1.6 (0.2, 3.0) c*	1.5 (0.1, 2.9)*	−0.2 (−0.3, 0.0)^c^**	−0.3 (−0.5, −0.1)*
Height (cm)	173.0 ± 6.9	6.9	−0.5 (−1.9, 0.9)	−1.4 (−3.0, 0.1)**	0.0 (−0.1, 0.2)	0.2 (0.0, 0.4)*
*Lifestyle*						
Smoking						
Never	36.2%		—	—	—	—
Past	60.9%		−3.4 (−6.3, −0.6)*	−3.8 (−6.7, −0.9)*	−0.6 (−1.0, −0.3)*	−0.6 (−0.9, −0.2)*
Current	2.9%		−7.5 (−15.7, 0.7)**	−7.4 (−15.6, 0.8)**	−0.7 (−1.7, 0.3)	−0.7 (−1.7, 0.3)
Caffeine intake (mg/day)	261.4 ± 259.3	259.3	−1.4 (−2.7, −0.0)*	−1.4 (−2.8, −0.0)*	−0.2 (−0.4, 0.0)*	−0.2 (−0.4, −0.0)*
Alcohol (drinks/week)	3.7 ± 6.8	6.8	−0.7 (−2.1, 0.6)	−0.8 (−2.2, 0.5)	−0.1 (−0.3, 0.1)	−0.1 (−0.3, 0.1)
Physical activity (PASE)	141.4 ± 65.9	65.9	0.0 (−1.4, 1.4)	0.2 (−1.2, 1.6)	0.2 (−0.0, 0.3)**	0.1 (−0.1, 0.3)
Walk for exercise (h/day)	1.8 ± 0.8	0.8	−0.9 (−2.3, 0.6)	−0.8 (−2.2, 0.7)	−0.0 (−0.2, 0.2)	−0.0 (−0.2, 0.2)
Supplemental calcium intake (mg/day)	278.3 ± 389.6	389.6	0.5 (−0.8, 1.9)	0.7 (−0.7, 2.0)	0.2 (0.0, 0.4)*	0.2 (0.0, 0.3)*
Total calcium (mg/day)	1126.7 ± 564.9	564.9	2.0 (0.6, 3.3)*	2.0 (0.7, 3.4)*	0.3 (0.1, 0.4)*	0.3 (0.1, 0.4)*
Daily vitamin intake (IU/day)	172.8 ± 123.2	123.2	1.2 (−0.2, 2.6)	1.2 (−0.2, 2.5)	0.0 (−0.1, 0.2)	0.1 (−0.1, 0.2)
*Medical history*						
Nontrauma fracture since age 50	15.3%		−7.1 (−10.9, −3.3)*	−7.2 (−10.9, −3.4)*	−1.0 (−1.4, −0.5)*	−0.9 (−1.4, −0.5)*
No diabetes	80.9%		−	−	−	−
Diabetes and non-TZD use	15.8%		3.0 (−0.8, 6.8)	3.9 (−1.3, 6.4)	−0.3 (−0.7, 0.2)	−0.1 (−0.6, 0.3)
Diabetes and TZD use	3.3%		10.8 (3.0, 18.4)*	9.8 (2.0, 17.6)*	−0.0 (−1.0, 0.9)	0.2 (−0.7, 1.2)
Hypertension	52.8%		5.1 (2.3, 7.8)*	4.8 (2.0, 7.5)*	0.3 (−0.0, 0.7)**	0.4 (0.1, 0.8)*
MI	17.1%		1.0 (−2.6, 4.7)	1.0 (−2.6, 4.7)	0.0 (−0.5, 0.5)	0.0 (−0.4, 0.5)
Stroke	6.9%		2.4 (−3.0, 7.9)	2.2 (−3.2, 7.7)	0.2 (−0.5, 0.9)	0.3 (−0.4, 1.0)
Cancer	30.3%		0.0 (−3.0, 3.0)	0.0 (−3.0, 3.0)	−0.2 (−0.5, 0.2)	−0.2 (−0.6, 0.2)
Osteoporosis	3.5%		−15.8 (−23.2, −8.5)*	−15.4 (−22.7, −8.0)*	−2.1 (−3.1, −1.2)*	−2.3 (−3.2, −1.4)*
Rheumatoid arthritis	6.1%		1.6 (−4.3, 7.5)	1.2 (−4.7, 7.1)	−0.6 (−1.3, 0.1)	−0.5 (−1.2, 0.2)
Osteoarthritis	22.5%		1.1 (−2.2, 4.4)	1.1 (−2.2, 4.3)	0.1 (−0.3, 0.5)	0.1 (−0.3, 0.5)
Hyperthyroid	2.5%		1.3 (−7.3, 10.0)	1.2 (−7.5, 9.8)	0.1 (−1.0, 1.2)	0.1 (−0.9, 1.2)
Hypothyroid	6.4%		2.0 (−3.5, 7.5)	2.1 (−3.4, 7.6)	0.1 (−0.6, 0.8)	0.1 (−0.6, 0.8)
COPD	10.1%		−4.0 (−8.5, 0.6)**	−4.1 (−8.6, 0.5)**	−0.5 (−1.1, 0.1)**	−0.5 (−1.1, 0.1)**
Parkinson's	1.1%		−12.7 (−27., 1.9)**	−12.5 (−27.1, 2.1)**	−0.8 (−2.6, 1.0)	−0.8 (−2.6, 1.0)
Gastrectomy	5.5%		−2.0 (−8.0, 3.9)	−2.0 (−7.9, 4.0)**	−0.6 (−1.4, 0.1)**	−0.7 (−1.5, −0.1)*
Kidney stones	13.3%		−3.2 (−7.2, 0.8)	−3.4 (−7.4, 0.7)	−0.1 (−0.6, 0.4)	−0.1 (−0.6, 0.4)
*Medications*						
Androgens	0.6%		6.8 (−10.7, 24.2)	5.7 (−11.8, 23.1)	2.8 (0.7, 5.0)*	3.1 (0.9, 5.2)*
Testosterone injections	0.5%		7.1 (−11.8, 25.9)	6.2 (−12.6, 25.0)	1.4 (−1.0, 3.7)	1.6 (−0.8, 3.9)
Antiandrogen use	0.5%		−8.1 (−27.0, 10.7)	−8.1 (7.7, −23.9)	−3.2 (−5.5, −0.9)*	−3.2 (−5.5, −0.9)*
Cox–2 inhibitors	2.0%		−4.9 (−14.6, 4.8)	−4.8 (−14.5, 4.9)	−0.1 (−1.3, 1.2)	−0.1 (−1.3, 1.1)
NSAID use	16.2%		−1.1 (−4.8, 2.6)	−1.5 (−5.2, 2.2)	−0.3 (−0.7, 0.2)	−0.2 (−0.6, 0.3)
Thiazide diuretic use	19.0%		3.3 (−0.2, 6.8)**	3.0 (−0.5, 6.5)**	0.3 (−0.1, 0.8)	0.4 (−0.0, 0.9)**
Nonthiazide diuretic use	11.8%		2.7 (−1.6, 7.0)	2.1 (−2.3, 6.4)	−0.1 (−0.6, 0.4)	0.0 (−0.5, 0.6)
Loop diuretics use	7.5%		2.5 (−2.8, 7.9)	1.7 (−3.7, 7.1)	−0.4 (−1.0, 0.3)	−0.2 (−0.8, 0.5)
Statins	45.2%		2.0 (−0.8, 4.8)	1.9 (−0.9, 4.6)	0.3 (−0.1, 0.6)	0.3 (−0.1, 0.6)
Nitrates	6.5%		2.3 (−3.3, 7.9)	2.2 (−3.4, 7.8)	0.6 (−0.1,1.3)	0.6 (−0.1, 1.3)
Beta blockers	33.3%		1.4 (−1.5, 4.3)	1.2 (−1.7, 4.1)	0.2 (−0.2,0.5)	0.2 (−0.1, 0.6)
Anticonvulsants	3.4%		1.6 (−6.0, 9.2)	1.6 (−6.0, 9.2)	−0.6 (−1.6, 0.3)	−0.6 (−1.6, 0.3)
SSRI use	4.3%		4.3 (−2.5, 11.1)	4.0 (−2.8, 10.8)	0.3 (−0.6, 1.1)	0.3 (−0.5, 1.2)
Tricyclic antidepressants	1.1%		1.0 (−11.8, 13.9)	0.1 (−12.7, 13.0)	1.1 (−0.5, 2.7)	1.3 (−0.3, 2.9)
Thyroid hormone use	8.4%		1.3 (−3.6, 6.3)	1.3 (−3.6, 6.3)	0.3 (−0.3, 0.9)	0.3 (−0.3, 0.9)
Corticosteroids (any)	9.3%		−3.5 (−8.2, 1.3)	−3.4 (−8.2, 1.3)	−0.5 (−1.1, 0.1)**	−0.6 (−1.1, 0.0)**
Bisphosphonates	3.6%		−20.8 (−28.1, −13.5)*	−20.2 (−27.6, −12.9)*	−1.6 (−2.5, −0.7)*	−1.8 (−2.7, −0.9)*
*General health*						
Health: Good/excellent	85.9%		0.0 (−4.0, 4.0)	0.3 (−3.7, 4.3)	0.6 (0.1, 1.1)*	0.5 (0.0, 1.0)*
*Neuromuscular*						
Gait speed (m/s)	1.2 ± 0.2	0.2	−0.2 (−1.7, 1.3)	0.1 (−1.4, 1.6)	0.4 (0.2, 0.6)*	0.4 (0.2, 0.6)*
Chair stands (s)	11.6 ± 3.4	3.4	0.2 (−1.3, 1.7)	−0.1 (−1.6, 1.5)	−0.3 (−0.5, −0.1)*	−0.2 (−0.4, −0.1)*
Stands with arms	9.0%		−1.1 (−6.0, 3.8)	−2.1 (−7.0, 2.8)	−0.9 (−1.5, −0.3)*	−0.7 (−1.3, −0.1)*
Grip strength (kg)	37.5 ± 7.7	7.7	−0.5 (−2.0, 1.0)	−0.8 (−2.3, 0.7)	0.3 (0.1, 0.5)*	0.3 (0.1, 0.5)*

a For continuous variables, the units approximate 1 SD; for dichotomous variables, the referent group does not have the characteristic.

b Effect of age alone.

c Effect of weight alone.

* *p* ≤ .05.

** .05 < *p* ≤ .1.

**Table 2:** Percent Difference in Trabecular And Cortical vBMD of the Tibia per Unit Change in Potential Correlates for Age- and Age- and Weight-Adjusted Models

			Percent difference in vBMD per unit change (95% CI)			
			
Variable	Mean ± SD or prevalence	Unit^a^	Age-adjusted trabecular vBMD	Age- and weight-adjusted trabecular vBMD	Age-adjusted cortical vBMD	Age-and weight-adjusted cortical vBMD
*Demographics*						
Age (years)	77.2 ± 5.1	5.1	−1.8 (−2.8, −0.8) b*	−1.3 (−2.4, −0.3)*	−0.4 (−0.6, −0.2)b,*	−0.5 (−0.7, −0.3)*
Race						
White	97.9%		—	—	—	—
African-American	1.4%		−7.0 (−15.9, 2.0)	−6.3 (−15.3, 2.7)	0.3 (−1.3, 1.9)	0.2 (−1.4, 1.7)
Asian	0.2%		−15.0 (−39.4, 9.5)	− 12.1 (−36.6, 12.3)	2.8 (−1.4, 7.0)	2.3 (−1.9, 6.5)
Hispanic	0.3%		−7.1 (−24.4, 10.3)	−7.7 (−24.9, 9.6)	−0.5 (−3.5, 2.5)	−0.4 (−3.3, 2.6)
Other	0.2%		7.6 (−16.8, 32.1)	7.5 (−16.9, 31.8)	4.4 (0.2, 8.6)*	4.5 (0.3, 8.7)*
>High school education	61.9%		2.7 (0.5, 4.7)*	2.7 (0.6, 4.8)*	0.5 (0.1, 0.8)*	0.6 (0.3, 1.0)*
*Site*						
Monongahela Valley, PA	53.9%		−0.6 (−1.5, 2.6)	−0.7 (−1.4, 2.7)	−0.9 (−1.2, −0.5)*	−0.9 (−1.2, −0.5)*
*Anthropometry*						
Weight (kg)	83.9 ± 13.5	13.5	1.7 (0.6, 2.8)^c^*	1.7 (0.6, 2.8)*	−0.3 (−0.5, −0.1)^c^*	−0.3 (−0.5, −0.1)*
Height (cm)	173.0 ± 6.9	6.9	−0.2 (−1.3, 0.8)	−1.3 (−2.4, −0.1)*	0.0 (−0.6, 0.2)	0.2 (0.0, 0.4)*
*Lifestyle*						
Smoking						
Never	36.2%		—	—	—	—
Past	60.9%		−3.0 (−5.2, −0.9)*	−3.4 (−5.5, −1.2)*	−0.5 (−0.9, −0.1)*	−0.5 (−0.8, −0.1)*
Current	2.9%		−5.6 (−11.9, 0.4) **	−5.7 (−11.8, 0.4)**	−0.6 (−1.6, 0.5)	−0.6 (−1.7, 0.4)
Caffeine intake (mg/day)	261.4 ± 259.3	259.3	−1.1 (−2.2, −0.1)*	−1.2 (−2.2, −0.2)*	−0.2 (−0.3, 0.0)**	−0.2 (−0.3, 0.0)**
Alcohol (drinks/week)	3.7 ± 6.8	6.8	−0.4 (−1.4, 0.6)	−0.6 (−1.6, 0.5)	−0.2 (−0.3, 0.0)**	−0.1 (−0.3, 0.0)
Physical activity (PASE)	141.4 ± 65.9	65.9	0.7 (−0.4, 1.7)	0.9 (−0.2, 1.9)	0.1 (−0.1, 0.3)	0.1 (−0.1, 0.3)
Walk for exercise (h/day)	1.8 ± 0.8	0.8	−0.4 (−1.5, 0.7)	−0.3 (−1.4, 0.8)	−0.01 (−0.2, 0.1)	−0.1 (−0.3, 0.1)
Supplemental calcium intake (mg/day)	278.3 ± 389.6	389.6	−0.0 (−1.0, 1.0)	0.1 (−0.9, 1.2)	0.3 (0.1, 0.5)*	0.3 (0.1, 0.5)*
Total calcium (mg/day)	1126.7 ± 564.9	564.9	1.1 (0.1, 2.1)*	1.2 (0.1, 2.3)*	0.3 (0.1, 0.5)*	0.3 (0.1, 0.5)*
Daily vitamin intake (IU/day)	172.8 ± 123.2	123.2	0.9 (−0.1, 1.9)**	0.9 (−0.1, 1.9)**	−0.0 (−0.2, 0.2)	−0.0 (−0.2, 0.2)
*Medical history*						
Nontrauma fracture since age 50	15.3%		−8.2 (−11.0, −5.3)*	−8.2 (−11.0, −5.4)*	−0.9 (−1.4, −0.4)*	−0.9 (−1.4, −0.4)*
No diabetes	80.9%		—	—	—	—
Diabetes and non-TZD use	15.8%		1.4 (−1.5, 4.2)	0.8 (−2.1, 3.6)	−3.8 (−0.8, 0.2)	−2.3 (0.2, −0.6)
Diabetes and TZD use	3.3%		9.2 (3.4, 15.0)*	7.9 (2.1, 13.8)*	−0.6 (−1.1, 0.9)	0.2 (−0.8, 1.2)
Hypertension	52.8%		2.7 (0.6, 4.7)*	2.4 (0.2, 4.3)*	0.1 (−0.2, 0.5)	0.2 (−0.2, 0.6)
MI	17.1%		0.2 (−2.5, 2.9)	0.3 (−2.5, 3.0)	−0.4 (−0.9, 0.1)**	−0.4 (−0.9, 0.1)**
Stroke	6.9%		−0.3 (−4.4, 3.7)	−0.5 (−4.6, 3.5)	0.2 (−0.5, 0.9)	0.3 (−0.4, 1.0)
Cancer	30.3%		0.3 (−0.2, 2.5)	0.4 (−0.5, 2.3)	0.1 (−0.3, 0.5)	0.1 (−0.3, 0.5)
Osteoporosis	3.5%		−14.0 (−19.5, −8.5)*	−13.5 (−19.0, −8.0)*	−2.0 (−3.0, −1.1)*	−2.3 (−3.2, −1.4)
Rheumatoid arthritis	6.1%		4.1 (−0.2, 8.4)**	3.7 (−0.6, 8.0)**	−0.2 (−0.9, 0.6)	−0.1 (−0.8, 0.7)
Osteoarthritis	22.5%		1.7 (−0.8, 4.1)	1.7 (−0.8, 4.1)	−0.1 (−0.5, 0.4)	−0.1 (−0.5, 0.4)
Hyperthyroid	2.5%		1.7 (−5.8, 8.6)	1.7 (−5.2, 8.5)	0.5 (−0.7, 1.7)	0.5 (−0.7, 1.7)
Hypothyroid	6.4%		−1.6 (−5.8, 2.5)	−1.5 (−5.7, 2.6)	0.3 (−0.4, 1.1)	0.3 (−0.4, 1.0)
COPD	10.1%		−2.1 (−5.5, 1.3)	−2.2 (−5.6, 1.2)	−0.4 (−0.9, 0.2)**	−0.4 (−0.9, 0.2)**
Parkinson's	1.1%		−5.2 (−14.9, 4.4)	−5.1 (−14.7, 4.5)	0.6 (−1.1, 2.2)	0.5 (−1.1, 2.2)
Gastrectomy	5.5%		−4.6 (−9.1, −0.2)*	−4.6 (−9.1, −0.2)*	−0.2 (−0.9, 0.6)	−0.2 (−1.0, 0.6)
Kidney stones	13.3%		−2.7 (−5.7, 0.3)**	−2.8 (−5.8, 0.2)**	−0.0 (−0.6, 0.5)	−0.0 (−0.5, 0.5)
*Medications*						
Androgens	0.6%		11.2 (−1.9, 24.3)**	10.0 (−3.1, 23.1)	2.3 (0.1, 4.6)*	2.6 (0.3, 4.8)*
Testosterone injections	0.5%		8.0 (−6.2, 22.1)	7.0 (−7.1, 21.1)	0.6 (−1.8, 3.1)	0.8 (−1.6, 3.3)
Antiandrogen use	0.5%		−28.7 (−44.1, −13.3)*	−28.6 (−44.0, −13.3)*	−3.2 (−5.8, −0.5)*	−3.2 (−5.8, −0.5)*
Cox-2 inhibitor use	2.0%		−4.0 (−11.4, 3.4)	−4.0 (−11.4, 3.4)	−0.4 (−1.7, 0.8)	−0.5 (−1.7, 0.8)
NSAID use	16.2%		0.8 (−2.0, 3.6)	0.4 (−2.4, 3.2)	−0.3 (−0.7, 0.2)	−0.2 (−0.7, 0.3)
Thiazide diuretic use	19.0%		2.6 (−0.1,5.2)**	2.1 (−0.5, 4.8)**	0.3 (−0.1, 0.8)**	0.4 (−0.1, 0.9)**
Nonthiazide diuretic use	11.8%		1.4 (−1.8, 4.6)	0.8 (−2.5, 4.0)	−0.5 (−1.0, 0.1)	−0.4 (−0.9, 0.2)
Loop diuretics use	7.5%		0.4 (−3.6, 4.4)	−0.4 (−4.5, 3.6)	−0.8 (−1.5, −0.2)*	−0.7 (−1.4, −0.0)*
Statins	45.2%		−0.7 (−2.7, 1.4)	−0.8 (−2.9, 1.3)	−0.2 (−0.5, 0.2)	−0.2 (−0.5, 0.2)
Nitrates	6.5%		0.3 (−3.9, 4.5)	0.2 (−3.9, 4.4)	−0.1 (−0.8, 0.7)	−0.1 (−0.8, 0.7)
Beta blockers	33.3%		0.3 (−1.9, 2.4)	−0.1 (−2.2, 2.1)	−0.0 (−0.4,0.3)	0.0 (−0.4, 0.4)
Anticonvulsants	3.4%		−1.2 (−6.7, 4.4)	−1.1 (−6.7, 4.4)	−0.1 (−1.0, 0.9)	−0.1 (−1.0, 0.9)
SSRI use	4.3%		3.5 (−1.6, 8.6)	3.2 (−1.9, 8.3)	0.3 (−0.6, 1.2)	0.4 (−0.5, 1.3)
Tricyclic antidepressants	1.1%		−1.8 (−12.2, 8.7)	−2.5 (−12.9, 8.0)	1.2 (−0.6, 3.0)	1.3 (−0.5, 3.1)
Thyroid hormone use	8.4%		−0.8 (−4.5, 2.9)	−0.8 (−4.7, 2.9)	0.4 (−0.3, 1.0)	0.3 (−0.3, 1.0)
Corticosteroid (any) use	9.3%		−2.4 (−5.9, 1.1)	−2.3 (−5.8, 1.2)	−0.3 (−0.9, 0.3)	−0.3 (−0.9, 0.3)
Bisphosphonates	3.6%		−15.6 (−20.9, −10.2)*	−14.9 (−20.3, −9.5)*	−1.3 (−2.3, −0.4)*	−1.5 (−2.5, −0.6)*
*General health*						
Health: Good/excellent	85.9%		2.9 (−0.0, 5.9)**	3.3 (0.4, 6.3)*	0.9 (0.4, 1.5)*	0.9 (0.4, 1.4)*
*Neuromuscular*						
Gait speed (m/s)	1.2 ± 0.2	0.2	0.5 (−0.6, 1.6)	0.8 (−0.3, 2.0)	0.4 (0.8, 0.6)*	0.3 (0.1, 0.5)*
Chair stands (s)	11.6 ± 3.4	3.4	−0.2 (−1.2, 1.0)	−0.5 (−1.6, 1.0)	−0.3 (−0.5, −0.1)*	−0.3 (−0.5, −0.1)*
Stands with arms	9.0%		−5.8 (−9.5, −2.1)*	−6.9 (−10.6, −3.8)*	−0.9 (−1.5, −0.3)*	−0.8 (−1.4, −0.1)*
Grip strength (kg)	37.5 ± 7.7	7.7	−0.2 (−1.4, 0.9)	−0.5 (−1.7, 0.6)	0.2 (−0.0, 0.4)**	0.2 (0.0, 4.3)*

a For continuous variables, the units approximate 1 SD; for dichotomous variables, the referent group does not have the characteristic.

b Effect of age alone.

c Effect of weight alone.

* *p* ≤ .0.

** .05 < *p* ≤ .1.

### Pairwise comparisons and trend for vBMD by age

**Fig. 1:**
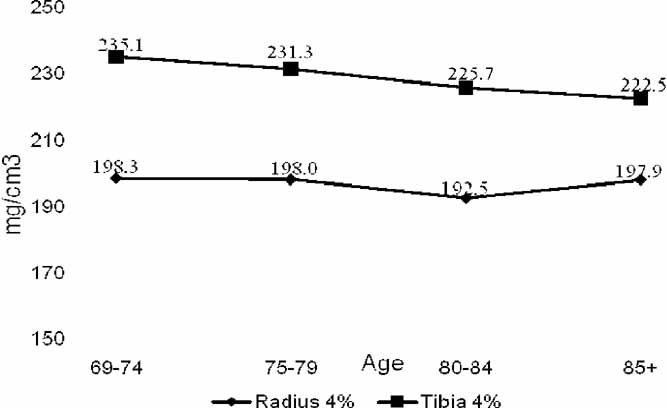
Trabecular vBMD by age group.

**Fig. 2:**
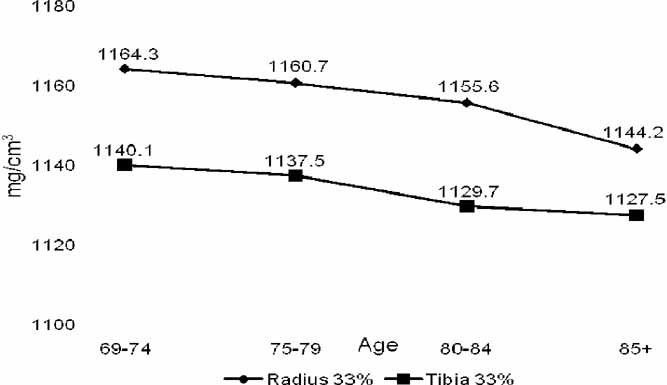
Cortical vBMD by age group.

Figures 1 and 2 show the unadjusted cross-sectional age-related patterns at each site. Trabecular vBMD at the radius did not differ by age (*p* = .483 for trend). The test of linear trend for tibial trabecular vBMD was statistically significant (*p* = .002). Participants aged 69 to 74 years had 4.1% and 5.7% greater trabecular vBMD at the tibia than those aged 80 to 84 years (*p* = .022) and 85 or more years (*p* = .030), respectively. Cortical vBMD varied by age at both skeletal sites (*p* < .001 for trend). At the radius, men aged 69 to 74 years had 0.7% and 1.8% greater cortical vBMD than those aged 80 to 84 years (*p* = .008) and 85 or more years (*p* < .001), respectively. Radial cortical vBMD was 1.5% greater among participants aged 75 to 79 years than men aged 85 or more years (*p* < .001). Also, men aged 80 to 84 years had 1.0% greater radial cortical vBMD than those aged 85or more years (*p* = .021). At the tibia, men aged 69 to 74 years had 0.9% and 1.1% greater cortical vBMD than those aged 80 to 84 years (*p* = .001) and 85 or more years (*p* = .005), respectively. Finally, those aged 75 to 79 years had 0.7% greater tibial cortical vBMD than men aged 80 to 84 years (*p* = .035).

### Multivariable models

The results of the multivariable analyses are summarized in Table 3. At the radius, multivariable models explained 4% and 9% of the overall variance for trabecular and cortical vBMD, respectively. The models for cortical and trabecular vBMD at the tibia site explained 8% and 10% of the overall variance. An increase of 1 SD (5.1 years) of age was significantly correlated with lower vBMD at all sites with the exception of trabecular vBMD at the radius. Having greater than a high school education was correlated with 3.0% greater trabecular vBMD of the tibia. Men from Monongahela Valley had −0.8% lower cortical vBMD than those from Minneapolis. A 1 SD (13.5 kg) increase in weight was associated with 2.3% greater tibial trabecular vBMD. However, this increase in weight was correlated with lower cortical vBMD of the radius (−0.5%) and tibia (−0.3%). A 1 SD (6.9 cm) increase in height was associated with lower trabecular vBMD at the tibia (−1.6%). A 1 SD (259.3 mg/day) increase in caffeine consumption was associated with lower cortical vBMD of the radius. Caffeine intake also was associated with lower trabecular and cortical vBMD of the tibia. A 1 SD (564.9 mg/day) increase in total calcium was associated with greater cortical and trabecular vBMD at both skeletal sites. Past smokers had significantly lower (−0.4%) cortical vBMD of the radius than individuals who had never smoked. History of a nontrauma fracture was associated with lower trabecular and cortical vBMD at both sites. Men who had diabetes and used TZD had greater trabecular vBMD of the radius (9.3%) and tibia (8.0%) than those with no diabetes. Having hypertension was correlated with greater (4.9%) trabecular and cortical (0.6%) vBMD of the radius. Rheumatoid arthritis was associated with a 5.3% greater trabecular vBMD of the tibia. Androgen use was correlated with greater cortical vBMD at both sites. Men who used antiandrogens had lower (−24.9%) trabecular vBMD of the tibia. An increase of 1 SD (7.7 kg) in grip strength was associated with greater (0.3%) cortical vBMD of the radius. Each 1 SD (3.4 seconds) increase in time to complete five chair stands was associated with lower (−0.3%) cortical vBMD at the tibia. Men who needed to use their arms to stand from a chair had lower (−7.2%) trabecular vBMD of the tibia.

**Table 3:** Percent Difference in vBMD per Unit Change of Each Correlate Using the Backward Elimination Procedure for Multiple Linear Regression Models

		Percent change in vBMD per unit change (95% CI)			
		
		Radius	Tibia	Radius	Tibia
					
Variable	Unit	Trabecular vBMD^a^	Trabecular vBMD^b^	Cortical vBMD^c^	Cortical vBMD^d^
*n*		1058	1065	1048	990
*Demographics*					
Age (years)	5.1	−0.6 (−1.9, 0.8)	−1.2 (−2.3, −0.1)	−0.4 (−0.6,−0.2)	−0.4 (−0.6, −0.2)
>High school education		—	3.0 (0.9,5.2)	—	—
Site (Monongahela Valley, PA)		—	—	—	−0.8 (−1.2, −0.4)
*Anthropometrics*				—	
Weight (kg)	13.5	—	2.3 (1.1, 3.6)	−0.5 (−0.7,−0.3)	−0.3 (−0.5, −0.1)
Height (m)	6.9	—	−1.6 (−2.8, −0.4)	—	—
*Lifestyle*					
Caffeine intake (mg/day)	259.3	—	−1.2 (−2.2, −0.2)	−0.2 (−0.4, −0.1)	−0.2 (−0.4, −0.1)
Total calcium (mg/day)	564.9	2.5 (1.1, 3.9)	1.5 (0.5, 2.6)	0.2 (0.1, 0.4)	0.2 (0.1, 0.4)
Past smoker		—	—	−0.4 (−0.8, −0.1)	—
*Medical history*					
Nontrauma fracture (any)		−7.2 (−11.1, −3.4)	−7.6 (−10.5, −4.8)	−0.8 (−1.3, −0.3)	−0.8 (−1.4, −0.3)
Hypertension		5.0 (2.2, 7.8)	—	0.6 (0.2, 0.9)	—
Diabetes and TZD use		9.3 (1.5, 17.0)	8.0 (2.2, 13.7)	—	—
Rheumatoid arthritis		—	5.3 (0.9, 9.6)	—	—
*Medications*					
Androgens		—	—	3.0 (0.9, 5.0)	2.7 (0.4, 5.0)
Antiandrogen		—	−24.9 (−43.9, −5.9)	—	—
*Neuromuscular function*					
Grip strength (kg)	7.7	—	—	0.3 (0.1, 0.5)	—
Chair stands (s)	3.4	—	—	—	−0.3 (−0.5, −0.1)
Stands with arms		—	−7.2 (−11.0, −3.4)	—	—
*r*^2^		0.04	0.10	0.09	0.08

a Total calcium intake (mg/day), nontrauma fracture (any), hypertension, and diabetes and TZD use were significant in the model.

b Age, weight, height, education, caffeine intake (mg/day), total calcium intake (mg/day), nontrauma fracture (any), diabetes and TZD use, rheumatoid arthritis, antiandrogens, and stands with arms were significant in the model.

c Age, weight (kg), caffeine intake (mg/day), total calcium intake (mg/day), past smoker, nontrauma fracture (any), hypertension, androgens, and grip strength (kg) were significant in the model.

d Age, weight (kg), site, caffeine intake (mg/day), total calcium intake (mg/day), nontrauma fracture (any), androgens, and time to complete five chair stands (s) were significant in the model.

### Secondary analyses

In the analysis that did not account for hypoglycemic medication use, men identified as having diabetes did not differ significantly by vBMD from men without diabetes. Insulin or hypoglycemic medication use without TZD use was not associated with vBMD. However, men who used TZDs had significantly greater trabecular vBMD of the radius (8.9%) and tibia (9.6%) than nonusers of antidiabetic medication. Finally, exclusion of nonwhites from the multivariable analysis did not change the correlates of vBMD that were identified at all sites.

## Discussion

We characterized demographic, anthropometric, lifestyle, and medical factors to understand their association with cortical and trabecular vBMD among older men. Unique features of this study include large sample size of men, comprehensive nature of the analyses, and ascertainment of correlates and outcome. Our study results suggest that some correlates of trabecular vBMD may differ from those of cortical vBMD.

Age was not associated with lower trabecular vBMD of the radius, consistent with the findings of a previous study by Dalzell and colleagues(20) but contrary to the findings of Riggs and colleagues,(21,22) Reduction in trabecular vBMD has been shown to occur before midlife (ages 20 to 49) in men and women and to continue unabated through life.(21–23) The mechanism for this early onset of trabecular vBMD loss remains unknown. A significant reduction in radial trabecular vBMD may have occurred at a younger age for this population. However, our results are cross-sectional comparisons across age groups, and longitudinal studies are needed. Consistent with prior findings, increasing age was associated with lower cortical vBMD.(20–22,24) In women, substantial cortical bone loss has been shown to occur after midlife in association with menopause and estrogen deficiency.(25) However, cortical bone loss in men seems to decrease at a constant rate in young adulthood until accelerating late in life.(22)

This study found a positive association between education and trabecular vBMD. This correlation has not been reported previously in men and may be a result of residual confounding. Higher education may lead to the adoption of a better lifestyle that could impact vBMD positively.

Body weight was associated with greater trabecular vBMD, consistent with two previous studies.(8,26) Several studies observed a positive association between weight and aBMD.(9,10,13) Larger skeletal frames and greater muscle and body fat in heavy individuals may increase the mechanical load on the skeleton, promoting mineralization and structural adaptive responses that can strengthen bone.(27) However, body weight was associated with lower cortical vBMD similar to two previous studies.(8,28) It is also possible that mechanical factors or differences in bone geometry may explain these findings. Paradoxically, increased mechanical loads can lead to bone microdamage, increased bone turnover, and a transient reduction in cortical vBMD.(29) Also, overweight individuals may adapt by increasing periosteal bone diameter, requiring lower cortical density to maintain the same strength.(30) Additional studies are needed to fully understand these relationships.

Similar to our findings, the Tobago Bone Health Study reported a negative association between height and trabecular vBMD, consistent with the negative association on femoral neck aBMD.(1,8) Taller individuals are known to experience greater height loss and have been shown to be at a higher risk of fracture than others.(31–33)

Several lifestyle and dietary factors were correlated with vBMD. Higher caffeine intake was associated with lower cortical and trabecular vBMD, similar to a previous study that examined calcaneus BMD in a young population of primarily white men.(26) However, some studies on aBMD in older white men(1,9,10) and another on vBMD in African men found no association.(8) Caffeine may reduce BMD by decreasing intestinal calcium absorption, but it also may be a marker for lower calcium consumption in our population (*r* = −0.1, *p* = .059).(34) Our study found that past smokers had significantly lower cortical vBMD. A study on men of African descent found a negative correlation among smokers for trabecular and cortical vBMD,(8) and another found a negative association among smokers for trochanter aBMD in a cohort of primarily elderly white men.(10) However, the Gothenburg Osteoporosis and Obesity Determinants (GOOD) failed to find a significant association between smoking and vBMD.(35) Smoking has been shown to have a negative effect on BMD by decreasing calcium absorption or by inhibiting the proliferation of osteoprogenitor cells.(36,37) Total calcium use was associated with greater trabecular and cortical vBMD, which is consistent with studies on aBMD(1,12,54) and vBMD(25) in white men and another in African men.(8) An adequate intake of calcium is needed to ameliorate the progressive loss of bone with age.(38)

History of fracture was one of the strongest factors related to lower trabecular and cortical vBMD at both skeletal sites and is consistent with studies of aBMD(1,13) and vBMD(23,39,40) in men. Diabetic men using TZD had greater trabecular vBMD at both skeletal sites. Greater weight among TZD users compared with diabetic men with no TZD use (95.3 versus 88.5 kg, *p* = .013) and nondiabetic men (95.3 versus 82.9 kg, *p* < .001) may have contributed to these findings. Prior research shows a negative association between TZD use and aBMD in diabetic men and women,(40,41) consistent with the observation that TZDs increase bone marrow adiposity, resulting in a decrease in osteoblastogenesis, and possibly affect the aromatase pathway, leading to a reduction in estrogen production.(42,43) Our findings are surprising and need to be replicated in other studies. Hypertension was associated with higher trabecular and cortical vBMD, comparable with previous studies for aBMD(1) in white men and vBMD in men of African heritage.(11) However, Orwoll and colleagues reported that hypertension was related to lower aBMD in a largely white population of men aged 60 years and older.(13) A positive association may arise from confounding by the use of certain medications such as thiazide (TZ) diuretics, which may increase BMD owing to their ability to improve calcium retention.(9,44,45) However, excluding TZ diuretic users did not change the association, making it likely that other factors were involved. Rheumatoid arthritis was unexpectedly associated with trabecular of the tibia and may have occurred by chance. An earlier report using the same cohort found no association between rheumatoid arthritis and aBMD,(1) while another study found a negative correlation among older white men.(13) Anti-inflammatory medications such as nonsteroidal anti-inflammatory drugs (NSAIDs) have been shown to increase BMD.(1,46) Excluding men who used NSAIDs attenuated the association, making it no longer significant.

Androgen-replacement therapy was associated with higher cortical vBMD at both skeletal sites, consistent with past findings on trabecular vBMD(47) and aBMD(48) at the lumbar spine. High androgen levels may help to maintain BMD by promoting osteoblast differentiation and inhibiting osteoclast recruitment.(49) The very low prevalence (0.6%) of androgen users likely contributed to the null finding for trabecular vBMD. Antiandrogen use was independently related to lower trabecular vBMD of the tibia. The Tobago Bone Health Study also found that antiandrogen users had significantly lower cortical vBMD at the radius and tibia.(8) Androgen-deprivation therapy or surgery to treat prostate cancer may result in hypogonadism, which is associated with lower BMD.(50,51)

The relationship between poor neuromuscular function and lower vBMD has been documented in several studies.(1,24,52) Although the mechanism for this association is not entirely clear, it may be due to the lower skeletal loading that occurs as a result of having weaker muscles.(53) Lower grip strength was associated with lower cortical vBMD at the radius, consistent with past reports on aBMD(1) and vBMD(23) in Japanese men. Our study also found a negative association between using arms to stand from a chair and lower trabecular vBMD, similar to a study in this cohort on aBMD.(1)

Our multivariable models explained up to 10% of the variance in trabecular vBMD and up to 9% of the variance in cortical vBMD. These estimates are slightly lower than reports on aBMD(1,9,13) but compare well with similar studies on vBMD(8,11,20,26) in men. A possible explanation for this observation may be that aBMD is a combination of density, size, and geometry of both cortical and trabecular bone, whereas vBMD in our study reflects the density of the voxels in the cortical or trabecular bone. These results suggest that there are unknown factors, possibly genetic, related to vBMD in older men that we have yet to identify. Fewer correlates were identified for trabecular vBMD of the radius when compared with tibial trabecular vBMD. The tibia is a major weight-bearing site compared with the radius, so variables such as weight and physical function may exert a greater influence on vBMD.

This study had some potential limitations. Based on the cross-sectional nature of this study design, causality cannot be established because we are unable to determine temporal relationships between the variables. Many of the variables were collected through self-report, so there was potential for recall bias resulting in misclassification. African Americans are known to have higher aBMD(1,55) and central vBMD(56) than whites. It is likely that the small number of African Americans (1.4%) in our sample reduced our potential to detect an association. Other findings also may have been affected by lower power (eg, current smokers: 2.9%). Despite statistical significance, the clinical significance of some of the findings is questionable owing to low percentage differences in vBMD.

In summary, we found that the correlates of trabecular and cortical vBMD differed in our large cohort of older men. Bones vary in their composition, with some made primarily of cortical bone and others that are almost entirely trabecular bone. An ascertainment of risk factors associated with trabecular and cortical vBMD may lead to better understanding and preventive efforts for osteoporosis in men. Our study also adds to the growing literature on the inverse association between body weight and cortical vBMD. Longitudinal studies are needed to better understand the mechanisms underlying these differential associations.
